# Preventing frailty with the support of a home-monitoring and communication platform among older adults—a study protocol for a randomised-controlled pilot study in Sweden

**DOI:** 10.1186/s40814-022-01147-4

**Published:** 2022-08-23

**Authors:** Minna Teriö, Rodrigo Pérez-Rodríguez, Tania Guevara Guevara, Myriam Valdes-Aragonés, Maksims Kornevs, Sanna Bjälevik-Chronan, Marina Taloyan, Sebastiaan Meijer, Susanne Guidetti

**Affiliations:** 1 Division of Occupational Therapy, Department of Neurobiology, Care Sciences and Society, Karolinska Institutet, Alfred Nobels Alle 23, B4, Huddinge, 141 83 Stockholm, Sweden; 2 Biomedical Research Foundation, Getafe University Hospital, Getafe, Spain; 3 Geriatrics Service, Getafe University Hospital, Getafe, Spain; 4 Department of Biomedical Engineering and Health Systems, KTH Royal Institute of Technology, Stockholm, Sweden; 5 Unit of Development/Social Care for Elderly, Enskede-Årsta-Vantörs City District, Stockholm, Sweden; 6 Division of Family Medicine and Primary Care, Department of Neurobiology, Care Sciences and Society, Karolinska Institutet, Academic Primary Healthcare Centre, Region Stockholm, Stockholm, Sweden; 7 Theme Women’s Health and Allied Health Professionals, Medical Unit Occupational Therapy and Physiotherapy, Karolinska University Hospital, Stockholm, Sweden

**Keywords:** Fragility, Frailty, Monitoring, Information and Communication Technology, ICT, eHealth, Healthcare, Disability, Prevention

## Abstract

**Background:**

POSITIVE (*i.e. maintaining and imPrOving the intrinSIc capaciTy Involving primary care and caregiVErs*) is a new intervention program consisting of home-monitoring equipment and a communication platform to support treatment of frailty symptoms initially in primary care and prevent disability in older adults.

**Methods:**

The primary objectives are to estimate the potential efficacy of the POSITIVE system on improving frailty in at least one point in Fried’s criteria and five points in Frailty Trait Scale. The secondary objectives are to (A) assess the recruitment, retention, drop-out rates, compliance with the intervention and the intervention mechanisms of impact; (B) evaluate the usability and acceptance of the POSITIVE system, and to get estimations on; (C) the potential efficacy of the intervention on improving the participants’ physical performance, cognitive functions, mood, independency level in activities in daily living, the impact on quality of life and number of falls during the follow-up period; (D) the impact on the caregiver quality of life and caregiver burden; and (E) on the consumption of health care resources, participants’ perception of health and level of care received, and healthcare professionals’ workload and satisfaction.

A randomised controlled, assessor-blinded pilot study design recruiting from a primary care centre in Stockholm Region will be conducted. Fifty older adults identified as pre-frail or frail will be randomised into a control or an intervention group. Both groups will receive a medical review, nutritional recommendations and Vivifrail physical exercise program. The intervention group will receive the POSITIVE-system including a tablet, the POSITIVE application and portable measurement devices. The participants receiving the POSITIVE program will be monitored remotely by a primary care nurse during a 6-month follow-up. Data will be collected at baseline, 3 and 6 months into the intervention though the platform, standardised assessments and surveys. A process evaluation as per Medical Research Council guidance will be conducted after the 6-month follow-up period.

**Discussion:**

The implications of the study are to provide estimations on the potential efficacy of the POSITIVE system in improving frailty among older adults and to provide relevant data to inform powered studies of potential efficacy and effectiveness, as well as to inform about the feasibility of the current study design.

**Trial registration:**

ClinicalTrials.gov. Registration number: NCT04592146. October 19, 2020.

**Supplementary Information:**

The online version contains supplementary material available at 10.1186/s40814-022-01147-4.

## Background

The world population is ageing rapidly. Ageing and age-related diseases pose a challenge for individuals, families as well as for social, economic, and healthcare systems. By 2050, the world population aged 60 years and older is expected to be over 2 billion people, up from 900 million in 2015 [1]. With the ageing process, several physiological changes occur and the risk of developing chronic diseases, disability, and dependency increase. Instead of focusing on the treatment of specific diseases, changes in the health systems are mandatory to prevent functional impairment and focus on guaranteeing healthy ageing [1].

Frailty is a health state defined as a progressive age-related decline in physiological systems that results in decreased reserves of intrinsic capacity, which confers extreme vulnerability to stressors and increases the risk of a range of adverse health outcomes. Frailty focuses on function and not specifically on diseases [2]. To date, several factors have been identified as risks of frailty among the community-dwelling older adults. In general, older age, female gender, low educational level, sedentary lifestyle, obesity, underweight, cognitive impairment, and comorbidities are associated with the development of frailty [3]. The estimated prevalence of frailty among older adults (+65 years) is 7–12% [4]. Frailty status can be assessed through standardised assessment tools, such as Linda Frieds’ Criteria and Frailty Trait Scale (FTS-5) [4, 5], where the first named measure components associated with frailty include unintentional weight loss, self-reported exhaustion, weakness (grip strength), slow walking speed, and low physical activity [4]. Frailty is potentially reversible with early screening and interventions including physical activity, a complete nutritional evaluation, reducing inappropriate drug prescription and preserving emotional vitality [6–11]. According to earlier research, physical exercise in combination with balanced nutrition has the strongest evidence level for positive outcomes in the treatment of frailty symptoms [10, 12].

Several studies have shown that telehealth can be effective in the treatment of different health conditions, and treatments supported by different information and communication technology (ICT) solutions lead to better health outcomes [13–16]. However, there is little knowledge on the impact of ICT-supported interventions in the treatment of frailty symptoms, due to few published studies on the topic [17, 18]. In this study, ICT will be used to prevent and treat frailty through a home-monitoring platform and a communication system between the patient, caregiver and health care personnel in primary care. The development of the new intervention POSITIVE system (i.e. *The Maintaining and imPrOving the intrinSIc capaciTy Involving Primary Care and caregiVErs*) emerged from the increasing need to develop innovative solutions to improve the delivery of health care for older age groups in Europe, but also to improve cost-effectiveness in treatments [19, 20].

The POSITIVE system consists of a set of applications running on mobile devices and involves all relevant actors in the care process: the older adult, his/her informal caregiver and the health care professionals [19]. The professional’s application further includes a communication platform and referral system between primary and specialized care. However, in this pilot study, the system will be adapted to fit the structure of health care delivery in Stockholm Region and will be used in primary care only.

The purpose of the home monitoring system is to collect relevant information of the frailty status, i.e. variables with high predictive power for adverse events including gait speed, power in the lower limbs, and involuntary weight loss [4], that is generated out of the clinical environment. It monitors the most relevant components associated with intrinsic capacity, such as functional level, nutritional- and emotional status of the older adults. By monitoring these components, the home monitoring kit can facilitate early detection of declines in health status [21] and thereby prevent disability and reduce costs in health care.

Through timely medical interventions, a physical exercise programme named Vivifrail (see Additional file 1), which is integrated into the POSITIVE system including several functions, older adults will potentially improve well-being and quality of life, reduce incidence of falls and hospitalisations and increase their feeling of safety in everyday life. The Vivifrail programme has proved to be efficient in improving the health status of older adults [22–24] and is one of the strategies in the European Union (EU) to promote healthy aging. However, in Sweden, the prescription of a physical exercise program targeting different levels of frailty (robust, pre-frail, frail and person with disability) as in Vivifrail, is a new concept. The Vivifrail programme is developed based on the idea that the health of the older adults can be measured according to their functional ability, instead of focusing on the treatment of separate diseases.

This protocol describes the procedures for the pilot study which will be conducted in Sweden as one of the cohorts within a multi-country POSITIVE project in Spain, Poland and Sweden. The protocol is in line with the Standard Protocol Items: Recommendations for Interventional Trials (SPIRIT) guideline [25] (SPIRIT Checklist see Additional file 2).

## Methods

The primary objectives of the study are to assess the potential of the POSITIVE system on improving frailty by at least one point in Fried’s criteria and five points in Frailty Trait Scale-5 (FTS-5). The secondary objectives are (A) to assess the recruitment, retention, drop-out rates, compliance with the intervention as well as the intervention mechanisms of impact; (B) to evaluate the usability and acceptance of the POSITIVE system, and to get estimations on; (C) the efficacy of the intervention on improving the participants’ physical performance, cognitive functions, mood, independency level in activities in daily living (ADL), quality of life and the impact on number of falls during the follow-up period; (D) the impact on the caregiver quality of life and caregiver burden; and (E) on the consumption of health care resources, participants’ perception of health and level of care received and healthcare professionals’ workload and satisfaction.

### Trial design

**Fig. 1:**
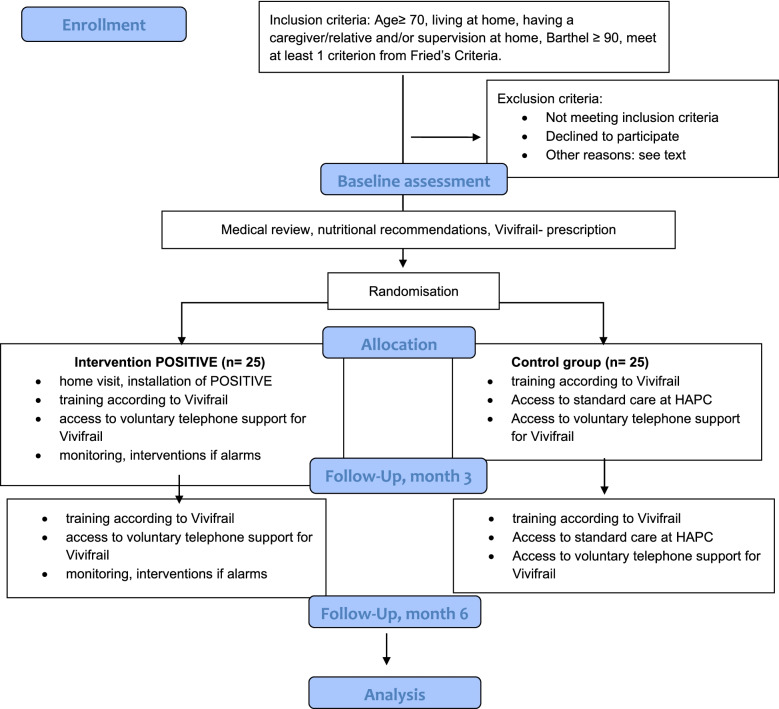
POSITIVE pilot study CONSORT-style flowchart

This is a randomised-controlled, single-blinded and prospective pilot study. The study processes are presented Fig. 1. Within the protocol, a process evaluation is scheduled and will be conducted in line with Medical Research Council (MRC) guidance for process evaluations. The components in the evaluation will follow the guidelines regarding the context, implementation level activities and mechanism of impact, such as participant’s responses to and interactions with the interventions [26].

### Study setting

This pilot study will be conducted at one primary care centre (PCC) at the Stockholm Region. In Sweden, the responsibility for delivering health care services is divided between the government, regions, and municipalities, and is for the most part funded by tax revenues. Municipalities have the responsibility for provision of older adults and primary care. Further, the basic care for all age groups is delivered by primary care. For care that requires specialist treatment and/or further investigation, primary care can refer patients to specialised care [27]. In the Stockholm Region, health care interventions to older adults can also be delivered in geriatric public or private in- and outpatient units, which specialise in acute and chronic conditions due to ageing. However, most patients who are treated in geriatric care are older adults with health conditions requiring acute interventions [20].

### Patient and public involvement

The POSITIVE-system has been developed using a user-centred design [28] including walk-throughs and testing demos among voluntary older adults in Spain and Sweden. These activities have proved to be crucial in the development of the system’s technical functions and user-friendliness. The results of the study will be disseminated to the study participants through the PCC.

### Sample size and power considerations

A sample size calculation is not required for feasibility and pilot studies [29, 30]. However, the sample needs to represent the target population and to be large enough to indicate the possible outcome of the intervention [31]. This study will recruit 50 participants, 25 in the control group (CG) and 25 in the intervention group (IG).

### Participants

The eligibility/exclusion criteria for study participation can be seen in Table 1.

**Table 1:** Eligibility and exclusion criteria

Eligibility criteria	Exclusion criteria
• Age ≥ 70 • Living at home • Having a caregiver/relative and/or supervision at home, or by a friend or partner, not necessarily living in the same building • Barthel Index ≥ 90 [29] • Have at least 1 criterion from Fried’s criteria	• Inadequate home infrastructure to host the required technology • Inability to understand how to use the POSITIVE system • Diseases that may affect prescription therapy: acute myocardial infarction in the last 3 months, unstable angina, aortic dissection, severe aortic stenosis, endocarditis/acute pericarditis, acute thromboembolic disease, acute heart failure, orthostatic hypotension (uncontrolled), recent fracture in the last month, terminal disease (< 12 months of life expectancy), other pathologies involving clinical instability • Psychiatric disorders that may potentially interfere in the clinical trial • History of alcohol/drugs abuse • Living with another participant, • Participating in other clinical studies • Three or more hospitalisations in the last year

To determine whether a participant can or cannot understand the use of the POSITIVE system, the participant will be asked to open the POSITIVE application in a tablet. Then the assessor will ask the participant to open different functions in the application and describe what they see. The caregivers’ and the health care workers’ perspectives on the POSITIVE system will be considered, and they will also be included as study participants.

### Participant timeline

**Fig. 2:**
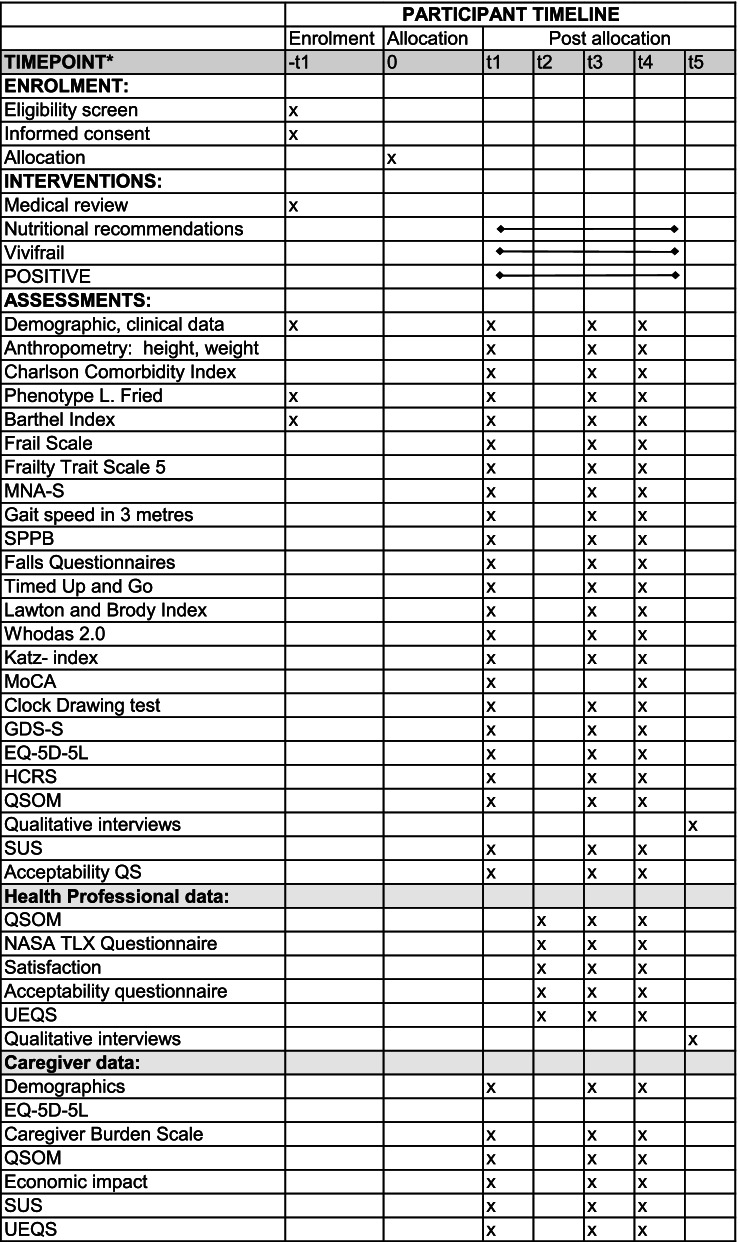
Schedule for enrolment, interventions and assessments. t-1, before allocation; t0, allocated; t1, baseline; t2, week 2; t3, week 12; t4, week 24; t5, post-follow-up. MNA-S, Mini Nutritional Assessment Short Version; SPPB, Short Physical Performance Battery; Whodas, World Health Organization Disability Assessment Schedule; MoCA, Montreal Cognitive Assessment; GDS-S, Geriatric Depression Scale Swedish Version; EQ-5D-5L, EuroQol- 5D-5L; HCRS, Health Care resources consumption; QSOM, Questionnaire on the Organisational Model; Acceptability questionnaires, Acceptability QS; SUS, System Usability Scale; UEQS, User Experience Questionnaire

Participant enrolment in Sweden started in November 2020, and the last follow-up is scheduled for October 2021. Time schedule for enrolment, interventions and assessments are shown in Fig. 2.

### Recruitment

A nurse at the PCC will pre-screen potential study participants from an electronic patient register system according to age and the excluding medical conditions. After identification, the nurse will send letters to potential participants, including an invitation to participate in the study. The potential participants will be asked to answer whether they are interested in taking part either by calling a contact number, answering through a letter or sending an e-mail. Posters and brochures about the study will be placed at the PCC and clinical staff will be asked to inform any potential older adult about the study. After receiving contact information for potential participants, a research assistant will call to inform them about the project and go through the eligibility criteria. If the participant meets the criteria, the research assistant will book a time for a home-visit. In this visit, the research assistant will provide oral and written information about the study and will ask for written consent. If the participant will meet all the eligibility criteria, and will obtain the written consent, he/she will be enrolled in the POSITIVE study. After the participant is enrolled, she/he will be randomised into the study.

### Randomisation

A computerised stratified randomisation technique will be applied in each of the study groups (CG and IG) based on age group (70–85, > 85), history of cognitive impairment and history of stroke to guarantee the existence of comparable groups. These factors have been significantly associated with frailty and pre-frailty status [32, 33]. To ensure objectiveness, the randomisation and allocation will be done by a person in the research team who will not be involved in the screening process.

### Interventions

#### Multimodal interventions

The participants will be randomised into one of the two groups and the interventions will be delivered in the participants’ homes. The duration of the intervention period is 6 months, at baseline, after 3 and 6 months into the intervention for both the control (CG) and the intervention group (IG). The time schedule for the study interventions is shown in Fig. 2.

Both the CG and the IG will receive a medical review via the PCC to ensure that the study participants follow appropriate medication. Furthermore, all participants will receive generic nutritional recommendations and physical exercise program according to the Vivifrail prescription guide [34]. Since the abovementioned interventions have evidence in improving frailty status [6–11], both treatment arms will receive these interventions, and this study can focus on the evaluation of the impact of using the POSITIVE technical solution to support the intervention.

The Vivifrail program is divided into six different exercise program levels, according to different functional levels and level of frailty, and possible risk of falls. The exercises include cardio exercise such as walking, strength and balance exercises and stretching. All participants will receive a program which is prescribed according to their functional level. In terms of improvement in the condition, a participant can continue to follow Vivifrail program according to a higher functional level or the program for robust persons until the end of the study. The programs and recommendations will be introduced and handed out by the research assistant at baseline. All participants will have the opportunity to call a physiotherapist in case they have questions or doubts considering the training according to Vivifrail. Furthermore, they can visit the PCC whenever they need during the follow-up, as a part of the standard care.

#### POSITIVE home monitoring system

**Fig. 3:**
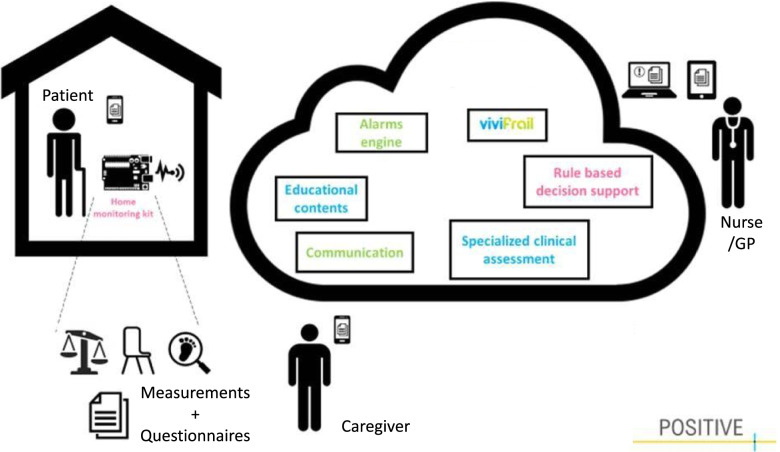
The POSITIVE system and its different components and users

The IG will receive the POSITIVE system (see Fig. 3), which will monitor the intrinsic capacity of the participants through a monitoring kit including physical tests and questionnaires, as well as compliance to the Vivifrail exercises, which are supposed to be performed at home. The interaction with the home monitoring system is handled by a mobile application that acts as a guiding element to the older person. The application works also as a data concentrator input point, not only enabling the older adult using the sensors, but also completing a set of questionnaires to enrich the information handled by the clinical professionals.

**Fig. 4:**
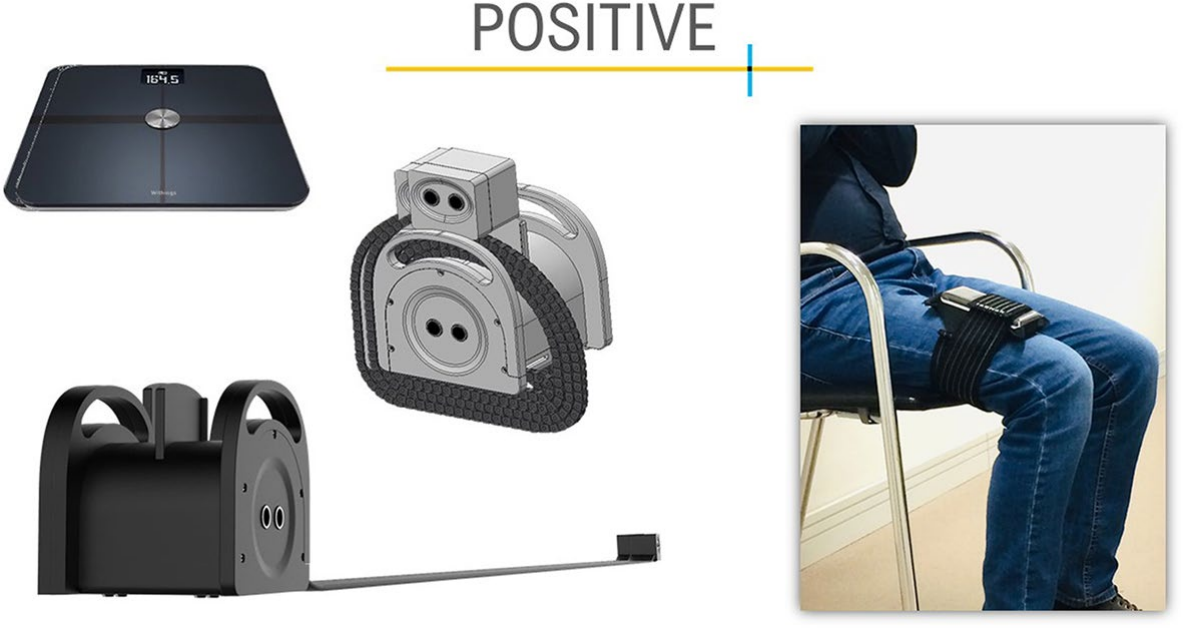
The POSITIVE mobile measurement devices

Results from physical tests will be automatically registered in the platform once the sensors send the data to the tablet via Bluetooth. These sensors include a gait speed placed on the floor (does not measure steps), a device to measure power in the lower limbs by monitoring the chair stand test, which will be placed on the thigh, and a smart weight scale to track involuntary weight loss (See Fig. 4).

Finally, adherence to the physical activity program is also measured by monitoring access through the app. The system will aim to prevent disability by triggering timely interventions if early decline in functional capacity is detected. Further, the system will provide nutritional recommendations depending on the participants’ health profile (generic, diabetic, obese, malnourished), which in turn can have an impact in vitality [21]. Additionally, the system will enable written communication between the older adult and a health care professional through the POSITIVE application.

The home monitoring system that belongs to the POSITIVE system will be installed and introduced by a researcher from the Royal Institute of Technology (KTH). The nurse connected to the PCC will remotely monitor the participants in the IG through the technology provided with the home monitoring system. During the 6-month follow-up period, if the system detects functional decline, the nurse will receive alerts from the system and will contact the participant to perform required interventions and, if needed, consult with the general practitioner (GP) responsible for the participants to plan interventions. The nurse will be recommended to consult the GP if she/he detects worsening in frailty status. The POSITIVE platform will even provide decision-making support for GPs and can give guidance on the need to refer the patient to specialised care. Further medical interventions required in primary care may include medical adjustments, updating the participants’ functional level in the POSITIVE platform and adjusting the Vivifrail program to their functional level, and referral of the patient to specialised care.

### Data collection

A technical researcher from KTH will collect data received from the POSITIVE platform. The server for the POSITIVE platform will be provided by Amazon Web Services. This service meets General Data Protection Regulation (GDPR) requirements [35] in terms of security and encryption of personal data, ability to restore the availability and access to personal data in a timely manner in the event of a physical or technical incidence. It also meets requirements for regularly testing, assessing and evaluating the effectiveness of technical and organizational measures for ensuring the security of processing. Further, the technology and methodology in the pilot meets security requirements, since pseudonymized data will be used. The pilot complies with the International Organization for Standardization (ISO) 27001 standards on information management security, and Hypertext Transfer Protocol Secure (HTTPS) is used for all connections.

The data collection in the periodic follow-ups will be conducted by a blinded research assistant. The data collection flow is shown in Fig. 1. The research assistant will write field notes after each contact with the participant to describe and understand the context.

The quantitative and qualitative data from the older adults and caregivers will be collected partly during home visits and partly through phone calls. Data from the informal caregivers will be collected through posted surveys if the caregivers do not live in the same household. Demographics from the participants and their caregivers, which will be collected consists of background data (age, comorbidities, type of housing, relationship to the older adult, employment, etc.). The participants’ health care resource consumption and their overall level or physical activity will be assessed at baseline, 3 and 6 months into the intervention (See Fig. 2).

Data collected from health care professionals will be collected through surveys and qualitative interviews at 2 weeks, 3 and 6 months into the intervention. The surveys consist of background data (age, education, number of working years, etc.) and structured questionnaires. The interviews will be conducted by a researcher focusing on the use of technology and the implementation processes.

All interviews conducted will be digitally recorded and transcribed verbatim. All identifying factors (e.g. names) will be deleted during transcription. Copies of the digital recordings will be destroyed after transcription has been completed. Interview transcriptions as well as quantitative data from the participants will be stored in the university’s database.

### Outcome measures

The different timepoints for the assessments are shown in Fig. 2.

#### Primary outcomes

The potential of the POSITIVE system on improving frailty will be assessed using *Linda Fried’s criteria* [4] and *FTS-5* [5] during a 6-month follow-up period. FTS-5 scores between 0 and 50. 0 is the best value and 50 the worst [5]. The two frailty scales correspond to the conceptual model of frailty phenotype, and in any case, the use of two scales could allow the performance of conducting a sensitivity analysis on the efficacy of the intervention on frailty measured in two different ways.

#### Secondary feasibility outcomes

Recruitment procedures, retention, and drop-out rates will be monitored continuously and registered throughout the study by the researchers. Compliance to the Vivifrail program for the IG will be evaluated through the registration in the physical exercise platform and for the CG through daily logbooks for exercises of the VIVIFRAIL guide. The influence of the context, the intervention level activities and the mechanisms of impact will be evaluated through monitoring process data, such as the level of daily use of the platform and conducting qualitative interviews with all participants from the IG (*n*=25) after the 6-month follow up, or until themes are saturated.

The usability and acceptance of the POSITIVE system will be asked using a questionnaire and will be collected by phone by the technical researcher from KTH.

Frailty transitions in prefrail and frail participants will be evaluated by *Fried’s Criteria* [4] and *FTS-5* [5]. The impact on locomotion in terms of changes in participants’ physical activity level and walking ability will be measured by *Short Physical Performance Battery (SPPB)* [36], *Gait Speed* [37] and *Timed Up and Go (TUG)* [38]. Participants’ independency level in ADL will be evaluated according to the *Barthel Index* [21, 31], *Katz-index* [39], *Lawton and Brody Index* [40] and *World Health Organisation Disability Assessment Schedule (WHODAS) 2.0* [41]. Given the inclusion criterion that establishes a Barthel index greater than 90, the target population is independent. Thereby in 6 months’ time, with the provided intervention, no modifications in the ADLs are likely to be observed. However, previous studies [21] demonstrate that this is sufficient time to see changes in the frailty status, which would imply, in case these changes are positive, a reduced risk to transition to disability.

The incidence of falls will be measured through *falls questionnaire* at baseline, which measures the number of falls pre-intervention, and 3 and 6 months into the intervention. The influence of the POSITIVE system on the cognitive functions will be measured by the *The Montreal Cognitive Assessment (MoCA)* [42] and the *clock drawing test* [43]. Changes in mood will be measured according to the *Geriatric Depression Scale (GDS- S)* [44]. Vitality will be addressed by measurements through *The Mini-Nutritional Assessment (MNA)* [45], and the instrument for assessment of the participant’s experienced quality of life will be *EuroQol-5D-5L* [46]. The participants’ perception of health and level of care received as well as involvement in care processes will be analysed by a questionnaire from *The International Consortium for Health Outcomes Measurement (ICHOM)*—Older person reference guide [47]. The usability and acceptance of the POSITIVE system will be evaluated by the *The System Usability Scale* (*SUS)* [48] and *Technology Acceptance Questionnaires*.

#### Caregivers and healthcare professionals

The caregiver’s quality of life and the caregiver burden will be measured by *EuroQol-5D-5L* [46] and *Caregiver Burden Scale* [49]. The economic impact on the caregiver will be investigated through an ad hoc questionnaire. The health care workers perspectives on the delivered intervention will be investigated by structured questionnaires related to the usability and acceptance of the technology and perspectives on the organisational model. The experienced workload will be measured by *the National Aeronautics and Space Administration Task Load Index (NASA TLX) questionnaire* [50].

### Data analysis

The potential efficacy of the POSITIVE-intervention will be analysed by an intention-to-treat method with a ‘per protocol analysis’, taking into consideration that the intervention group will be formed by those who do not have major technical problems, since this approach will portray better the use of the intervention in usual conditions at home. The participants will be analyed at different follow-up points to have information on some of those who will drop out later in the intervention process. For each follow-up visit, also the descriptive data will be presented as means and percentages. Linear and logistic mixed effects models will be used to assess the efficacy of the intervention and its impact on the measured parameters, to evaluate whether the objectives of the study are met or not. The one-tailed significance level will be set at *α*=0.05.

The qualitative data analysis will be performed from line-by-line transcription of the audios. The transcriptions will be coded by two researchers, and the data will then be merged into larger thematic categories, which will be discussed and compared by the researchers. The qualitative analysis will follow the content analysis process [51]. Results of the qualitative analysis will be reported at group level and individually, but information from individuals will not be identifiable. Comparisons between groups will be carried out.

## Discussion

The implications of the study are to provide estimations on the efficacy of the POSITIVE system in reducing frailty symptoms among older adults, prevent disability and dependency, as well as to affect several other health-related domains. Further, the study will contribute knowledge about the feasibility of the current study design in Sweden, perceived value of the POSITIVE system, its utility and the acceptability of the POSITIVE system among the different stakeholders. Thus, the study will give relevant data to inform powered studies of efficacy and effectiveness.

Our primary objective relates to improving frailty in at least one criterion. The multicomponent intervention addresses frailty as a clinical entity, measured with the Linda Fried’s criteria and with FTS-5. The multicomponent intervention we administer is known to be effective, but we are also exploring whether the support of our technology boots its effect. The two frailty scales have common items, such as grip strength, gait speed and level of physical activity, and we do not expect to see great differences between the scales. In the case we find any differences during the data analysis, we will discuss the possible effects of the intervention on the items that are not repeated, including weight loss and exhaustion in the case of the Fried’s scale, and balance and body mass index in the case of the FTS-5. Further, we will discuss the suitability of the different assessment tools to be used in future powered studies.

Limitations in this study include the challenges in blinding the assessor, since the study is not blinded for the participants, and they may reveal their allocated group to the assessor. However, the participants will be reminded in all contacts with the assessor not to reveal whether they are using the POSITIVE system or not. Another limitation is that the selection process of the participants will not be randomised, but the potential participants will be invited to contact the research group voluntarily if they are interested in the study. This fact may lead to selection bias considering that the study participants may not provide a heterogenous and representative sample of the older adult population in Sweden.

Lastly, even though the aim in this study is to have a cohort comparable to that of Spain and Poland which are part of the main POSITIVE project, the differences in the organisation of the health care system causes certain differences in the study processes and in the clinical praxis, and these should be considered when doing cross-cohort analysis in the main project.

Possible practical issues during the study may involve technical aspects, since many older adults may not be familiar with how to handle the technical equipment. However, a researcher from KTH will provide participants with support during the whole study, if needed. Another issue which may affect the study processes and participant recruitment is the current pandemic situation in the world, which in turn may negatively affect the recruitment of participants.

## Supplementary Information

**Additional file 1.** Vivifrail exercises.

**Additional file 2.** Description of the protocol according to SPIRIT checklist.

## Data Availability

The datasets used and/or analysed during the current study, as well as questionnaires and interview guides are available from the corresponding author on reasonable request.

## Abbreviations

ADL: Activities in daily life
CG: Control group
FTS: Frailty Trait Scale
GP: General practitioner
GDPR: General Data Protection Regulation
GDS-S: Geriatric Depression Scale—Swedish version
HCRS: Health care resources consumption
ICT: Information and Communication Technology
IG: Intervention group
ISO: International Organization for Standardization
KI: Karolinska Institute
KTH: The Royal School of Technology
LAR: Legally acceptable representative
MNA-S: Mini Nutritional Assessment Short Version
MoCA: Montreal Cognitive Assessment
MRC: Medical Research Council
NASA-TLX: National Aeronautics and Space Administration Task Load Index
PCC: Primary Care Centre
POSITIVE: Maintaining and Improving the Intrinsic Capacity Involving Primary Care and Caregivers
QS: Questionnaires
QSOM: Questionnaire on the Organisational Model
SPIRIT: Standard Protocol Items: Recommendations for Interventional Trials
SPPB: Short Physical Performance Battery
SUS: System Usability Scale
UEQS: User Experience Questionnaire
WHO: World Health Organization
WHODAS: World Health Organization Disability Assessment Schedule
