# Supplementary material for: Preventing frailty with the support of a home-monitoring and communication platform among older adults—a study protocol for a randomised-controlled pilot study in Sweden
Source: Pilot Feasibility Stud. 2022 Aug 23;8:190. doi: 10.1186/s40814-022-01147-4 (PMC9396907; doi:10.1186/s40814-022-01147-4)
Supplement: Supplementary file 1 — Additional file 1. Vivifrail exercises. [file 40814_2022_1147_MOESM1_ESM.pdf]

A

EXERCISE WHEEL

WALK

BEGIN THIS EXERCISE ONLY WHEN YOU HAVE IMPROVED YOUR MUSCLE STRENGTH.

5-10 SECONDS 5 SETS.

INCREASE THE TIME UNTIL YOU CAN WALK 1-2 MINUTES WITHOUT STOPPING.

REST

REMEMBER TO REST BETWEEN SETS.

BREATHE CORRECTLY

BREATHE NORMALLY, DO NOT HOLD YOUR BREATH DURING THE EXERCISES.

EXERCISES

IF THERE IS ANY DOUBT ABOUT HOW TO CARRY OUT THE EXERCI-SES, PLEASE CONSULT THE PASSPORT.

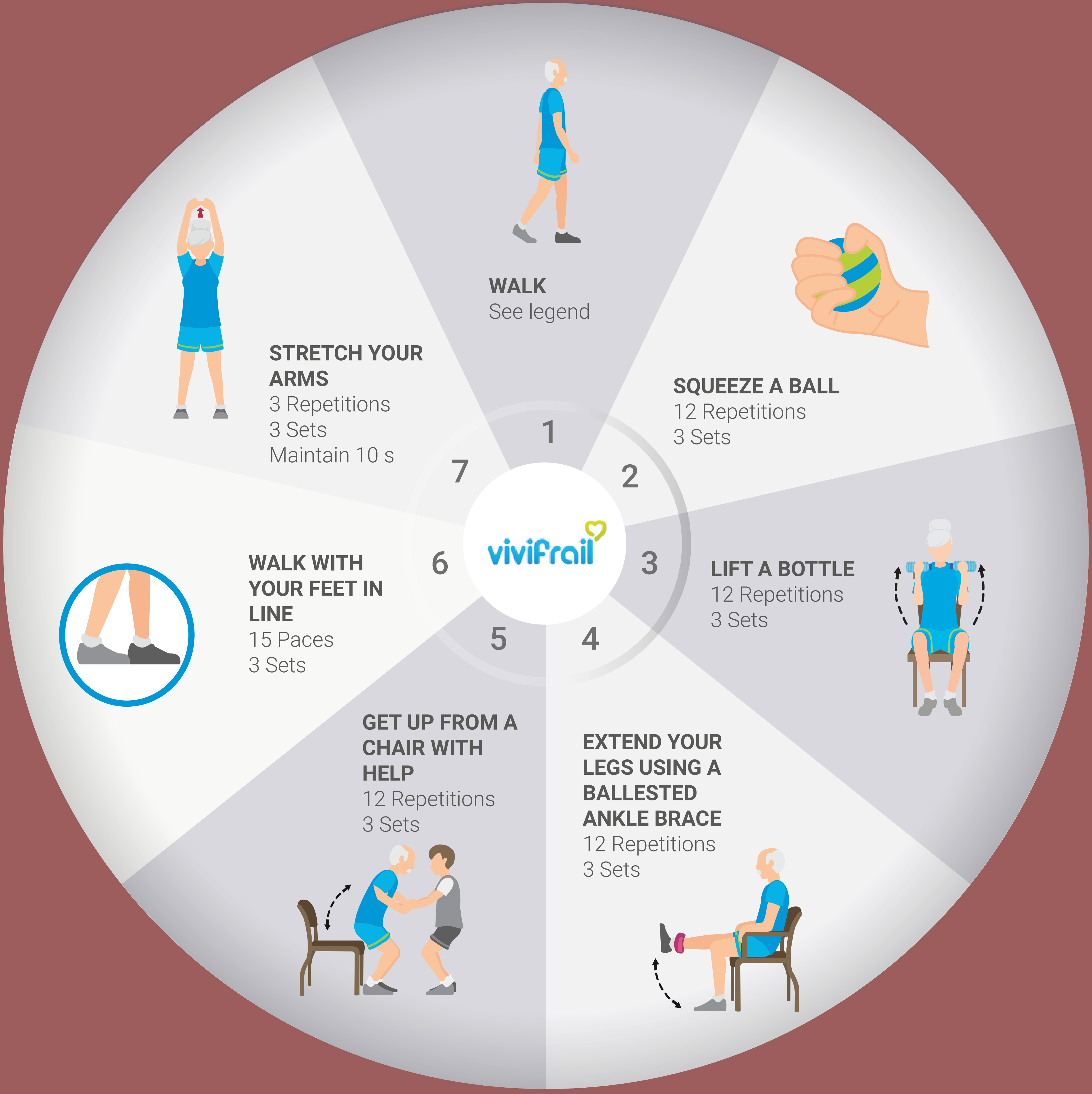

The project has been 65% cofinanced by the European Regional Development Fund (ERDF) through the Interreg V-A Spain-France-Andorra programme (POCTEFA 2014-2020). POCTEFA aims to reinforcthe economic and social integration of the French–Spanish–Andorran border. Its support is focused on developing economic, social and environmental cross-border activities through joint strategies favouring sustainable territorial development.

Multicomponent physical exercise program to prevent frailty and the risk of falls. © Mikel Izquierdo

B

EXERCISE WHEEL

WALK

2 MINUTES 5 SETS.

WALK AT A PACE THAT ALLOWS YOU TO KEEP A CONVERSATION CONTINUOUSLY, BUT THAT COSTS YOU A LITTLE EFFORT.

FROM WEEK 7, WALK 3 SETS OF 8 MINUTES.

REST

REMEMBER TO REST BETWEEN SETS.

BREATHE CORRECTLY

BREATHE NORMALLY, DO NOT HOLD YOUR BREATH DURING THE EXERCISES.

EXERCISES

IF THERE IS ANY DOUBT ABOUT HOW TO CARRY OUT THE EXERCISES, PLEASE CONSULT THE PASSPORT.

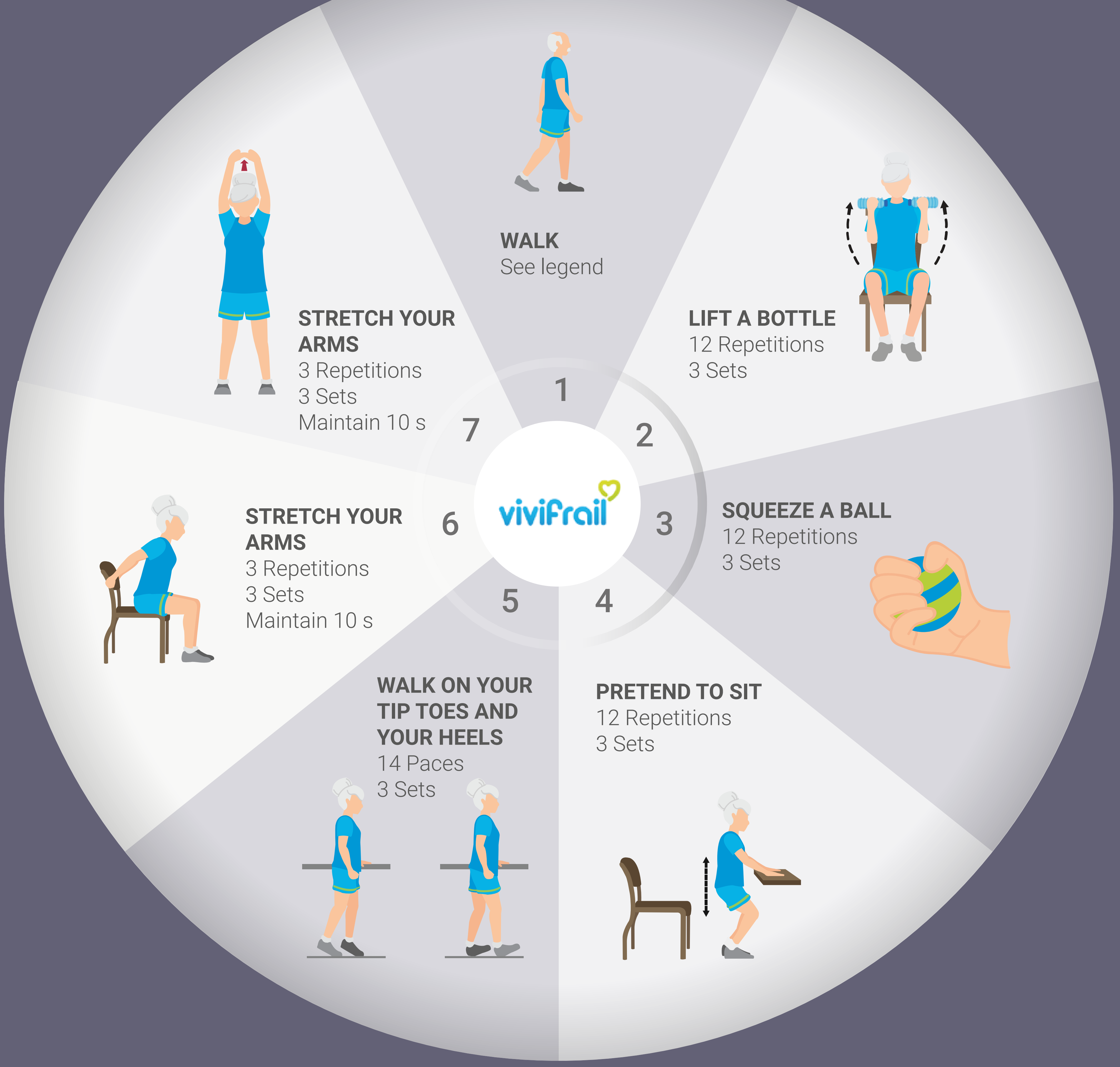

The project has been 65% cofinanced by the European Regional Development Fund (ERDF) through the Interreg V-A Spain-France-Andorra programme (POCTEFA 2014-2020). POCTEFA aims to reinforcthe economic and social integration of the French–Spanish–Andorran border. Its support is focused on developing economic, social and environmental cross-border activities through joint strategies favouring sustainable territorial development.

Multicomponent physical exercise program to prevent frailty and the risk of falls. © Mikel Izquierdo

C

EXERCISE WHEEL

WALK

110 MINUTES 3 SETS.

WALK AT A PACE THAT ALLOWS YOU TO KEEP A CON-VERSATION CONTINUOUSLY, BUT THAT COSTS YOU A LITTLE EFFORT.

FROM WEEK 7, WALK 3 SETS OF 15 MINUTES.

REST

REMEMBER TO REST FOR 2 MINUTES BETWEEN SETS.

BREATHE CORRECTLY

BREATHE NORMALLY, DO NOT HOLD YOUR BREATH DURING THE EXERCISES.

EXERCISES

IF THERE IS ANY DOUBT ABOUT HOW TO CARRY OUT THE EXERCISES, PLEASE CONSULT THE PASSPORT.

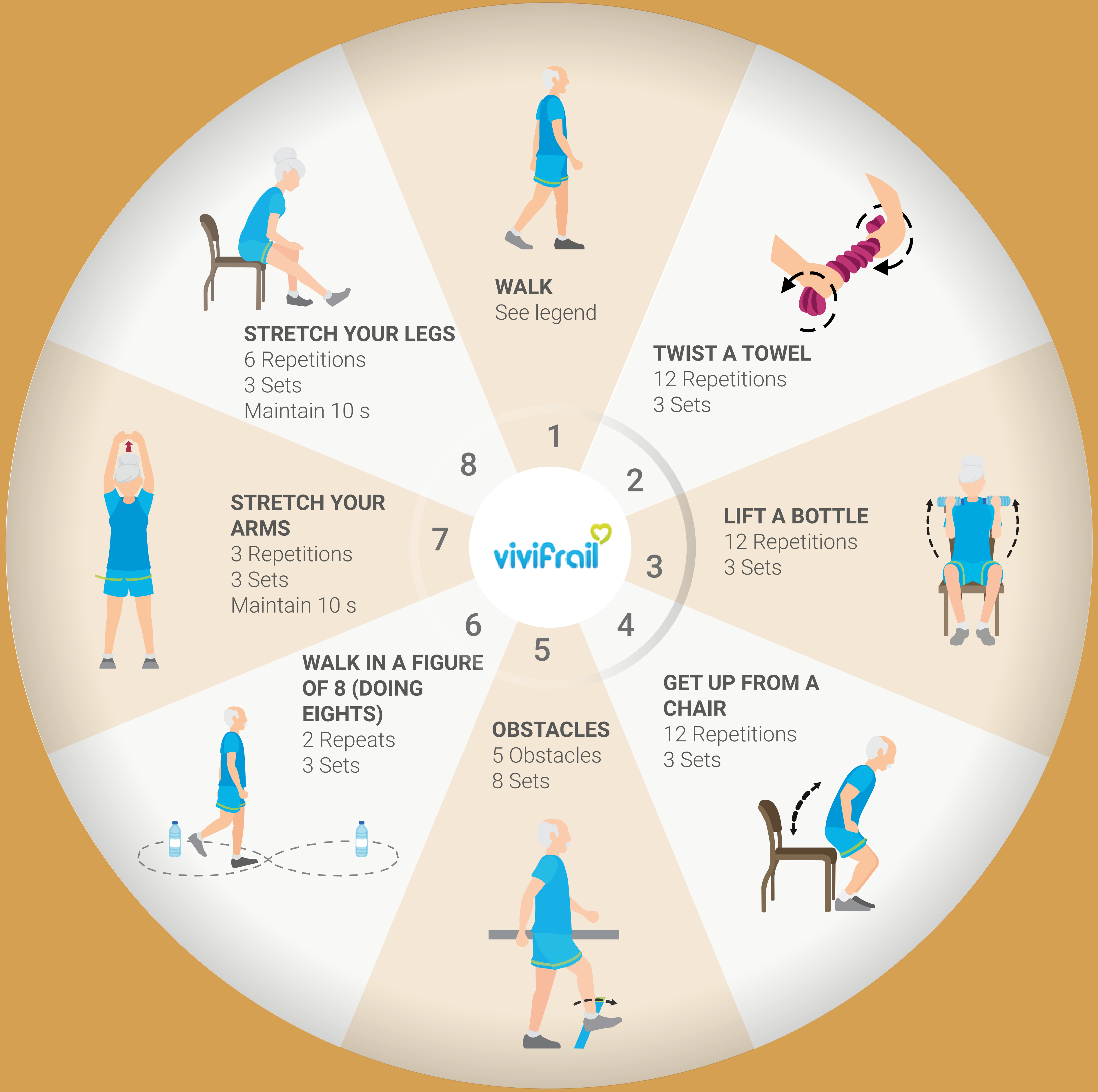

The project has been 65% cofinanced by the European Regional Development Fund (ERDF) through the Interreg V-A Spain-France-Andorra programme (POCTEFA 2014-2020). POCTEFA aims to reinforcthe economic and social integration of the French–Spanish–Andorran border. Its support is focused on developing economic, social and environmental cross-border activities through joint strategies favouring sustainable territorial development.

Multicomponent physical exercise program to prevent frailty and the risk of falls. © Mikel Izquierdo

D

EXERCISE WHEEL

WALK

20 MINUTES 2 SETS.

WALK AT A PACE THAT ALLOWS YOU TO KEEP A CON-VERSATION CONTINUOUSLY, BUT THAT COSTS YOU A LITTLE EFFORT.

FROM WEEK 7, WALK CONTINUOUSLY BETWEEN 30 & 45 MINUTES.

REST

REMEMBER TO REST FOR 2 MINUTES BETWEEN SETS.

BREATHE CORRECTLY

BREATHE NORMALLY, DO NOT HOLD YOUR BREATH DURING THE EXERCISES.

EXERCISES

IF THERE IS ANY DOUBT ABOUT HOW TO CARRY OUT THE EXERCISES, PLEASE CONSULT THE PASSPORT.

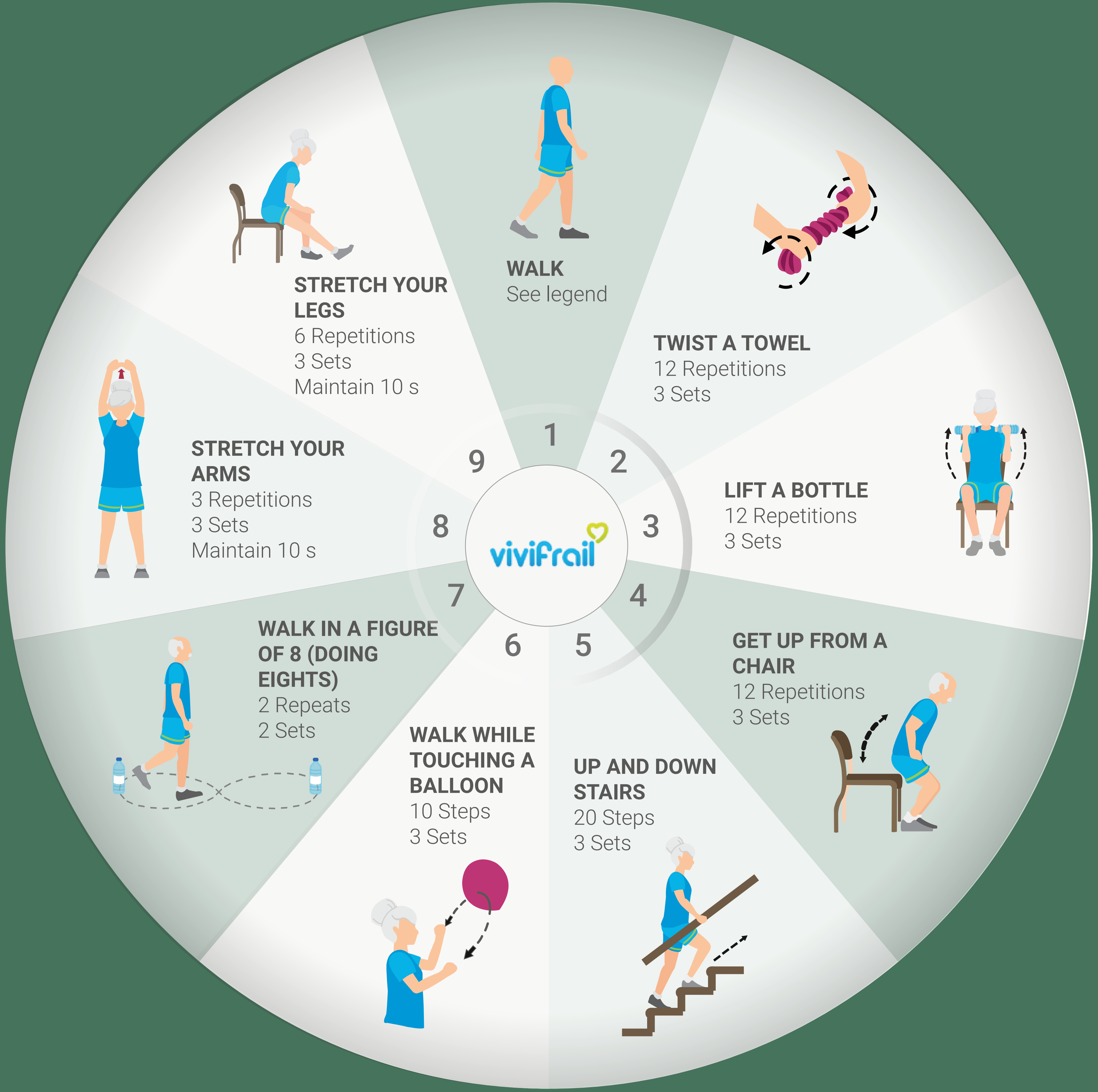

The project has been 65% cofinanced by the European Regional Development Fund (ERDF) through the Interreg V-A Spain-France-Andorra programme (POCTEFA 2014-2020). POCTEFA aims to reinforcthe economic and social integration of the French–Spanish–Andorran border. Its support is focused on developing economic, social and environmental cross-border activities through joint strategies favouring sustainable territorial development.

Multicomponent physical exercise program to prevent frailty and the risk of falls. © Mikel Izquierdo
